# Recent Advancements in Nanoparticle-Based Optical Biosensors for Circulating Cancer Biomarkers

**DOI:** 10.3390/ma14061339

**Published:** 2021-03-10

**Authors:** Chaima Amri, Arvind Kumar Shukla, Jin-Ho Lee

**Affiliations:** 1 Department of Convergence Medical Sciences, School of Medicine, Pusan National University, Yangsan 50612, Korea; a.chaima@pusan.ac.kr; 2 School of Biomedical Convergence Engineering, Pusan National University, Yangsan 50612, Korea; arvindkumarshukla@pusan.ac.kr

**Keywords:** optical biosensors, circulating cancer biomarkers, optical transduction, metal nanoparticles (M-NPs), quantum dots (QDs), upconversion nanoparticles (UCNPs)

## Abstract

The effectiveness of cancer treatment strongly depends on the early detection of the disease. Currently, the most common diagnostic method, tissue biopsy, takes time and can be damaging to the patient. Circulating cancer biomarkers such as circulating tumor DNA, micro-RNA (miRNA), tumor proteins, exosomes, and circulating tumor cells have repeatedly demonstrated their viability as targets for minimally invasive cancer detection through liquid biopsies. However, among other things, achieving a great sensitivity of detection is still challenging due to the very low concentration of biomarkers in fluid samples. This review will discuss how the recent advances in nanoparticle-based biosensors are overcoming these practical difficulties. This report will be focusing mainly on optical transduction mechanisms of metal nanoparticles (M-NPs), quantum dots (QDs), and upconversion nanoparticles (UCNPs).

## 1. Introduction

The early detection of cancer considerably impacts the effectiveness of oncotherapy. Currently, tissue biopsies are commonly used as an affordable and accurate diagnostic method. However, on top of being a time-consuming procedure, tissue biopsies can be difficult to reproduce. In certain cases, tissue sampling can require a more invasive procedure that can be frightening or even damaging to the patient. Thus, it is important to develop faster, less invasive, and more precise biosensors. Recently, the sensing of circulating cancer biomarkers such as circulating tumor DNA (ctDNA), circulating micro-RNA (miRNA), tumor proteins, exosomes, or even circulating tumor cells (CTCs) have been gaining a lot of attention as they allow for minimally invasive detection methods. However, the low concentration of those biomarkers renders most standard biosensors obsolete.

Circulating cancer biomarkers are molecules of different forms mostly present in body fluids such as the serum/plasma, saliva, or urine of cancer patients. Among those biomarkers, ctDNAs are short fragments of cell-free DNA originating from tumor cells [1]. The release mechanism of ctDNAs is not clearly understood yet, but recent studies have demonstrated a positive correlation between ctDNA levels and tumor burden in animal models [2]. On the other hand, circulating miRNA, stable non-coding small RNAs, are differentially expressed depending on the stage of tumor progression [3]. Currently, most ctDNA and miRNA analyses are performed in liquid biopsies through variations of polymerase chain reaction (PCR), microarray, or next-generation sequencing with each certain disadvantages: a small number of target genes, a low throughput, or the cost of the equipment [2,4,5,6,7]. Furthermore, serum proteins have been successfully targeted in the detection of various types of cancers such as breast cancer [8] and epithelial ovarian cancer [9], as their differential secretion is thought to be an indicator of cancer cells. Exosomes, nanosized molecular and genetic cargoes, have also demonstrated an excellent diagnostic ability as their main function is to act as signaling vesicles between cancer cells and their environment [10,11]. Last but not least, on a microscopic scale, circulating tumor cells are key players in cancer metastasis which makes them excellent diagnostic and prognostic biomarkers [12]. As circulating cancer biomarker expression is directly related to the presence of cancer cells, they offer a promising alternative to tissue biopsies. However, extracting a detectable amount of biomarkers, processing them without disturbing their integrity, targeting multiple biomarkers at the same time, and doing so in a timely manner are only a few of the many challenges faced when using the traditional techniques for the detection of circulating cancer biomarkers. To overcome these problems research is turning to nanoparticle-based biosensing.

**Figure 1:**
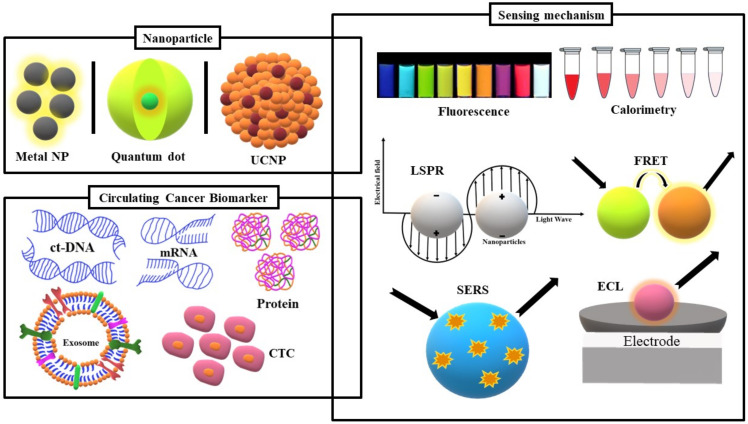
Schematic representation of the nanoparticles and the main sensing mechanisms of circulating cancer biomarkers covered in this review.

Of all the transduction mechanisms of nanoparticle-based biosensors, optical transduction, the core of this review, is the most preeminent in biosensing due to the speed and ease of its signal detection. Additionally, despite the existence of different types of nanoparticles, only a few demonstrate exceptional optical proprieties relevant to the subject matter. With that in mind, the focus of this review will be on the recent innovations in circulating cancer biomarkers optical nanobiosensors based on quantum dots (QDs), metal nanoparticles, and upconversion nanoparticles (Figure 1). The application of QDs in fluorescence and electrochemiluminescence (ECL) assays as well as their adaptation in fluorescence resonance energy transfer (FRET)-based sensors will be discussed. As metal nanoparticles (NPs) are not characteristically fluorescent, the focus will be on the effect of their localized surface plasmon resonance (LSPR) in optical transduction mechanisms such as colorimetry, surface-enhanced Raman scattering (SERS), and metal enhanced fluorescence (MEF). Finally, recent advances in upconversion nanoparticles (UCNPs)-based biosensors will also be covered.

## 2. Quantum Dots-Based Optical Biosensor for Circulating Cancer Biomarkers

Quantum dots are 2–10 nm luminescent semiconductor nanocrystals that possess size-tunable photophysical properties with a broad absorbance and a narrow emission that are very pertinent to the biosensing field [13]. This gives QDs a unique multiplex feature that can be used in the detection of multiples analytes at once [14]. They can be produced through different processes mainly divided into two categories: the top-down approach that consists of thinning a semi-conductor to achieve nanometer-sized particles and the bottom-up method where the self-assembly of QDs is promoted through nucleation or atom-by-atom layering [15]. QD cores and/or shells are typically made of elements from the groups II–VI: CdSe, CdS, CdTe, ZnSe, ZnS, ZnTe, HgS, HgSe, HgTe, and ZnO [16]. However, other elements such as carbon, graphene, and molybdenum oxide have been gaining more attention. The core-shell structure of the QD is another important feature of biosensing as the shell can be modified to allow for its bioconjugation with DNA, aptamers, and antibodies [17].

Since their first discovery, quantum dots have been frequently used as fluorophores to sense circulating cancer markers. In a CTC quantification assay, Min et al. combined the fluorescence of QDs with a magnetic bead-based isolation assay [18]. Briefly, QDs were functionalized with anti-epithelial cell adhesion molecule (EpCAM) antibodies; EpCAM is highly expressed in tumor cells. The nanoprobes were then added to a cell suspension of SK-Br3, a breast cancer cell line [18]. After removal of the excess QDs by centrifugation, anti-IgG-modified magnetic beads were incorporated and bound to the remaining anti-EpCAM antibodies. Then, a magnetic field was used to isolate the anti-EpCAM-QDs attached SK-Br3 cells. Measuring the fluorescence intensity of the isolated QDs with a plate reader allowed for the quantification of captured CTCs with an efficiency of 70–80% in 50 min [18]. In a similar experiment with a higher capture efficiency of about 90% in whole blood samples, aptamer EpCAM receptors were conjugated with graphene QDs (GQDs) to synthesize a “turn-on” biosensor based on nanosurface energy transfer (NSET) [19]. By using aptamers@magnetic Fe_3_O_4_ nanoparticle-bound GQDs as energy donors and MoS2 nanosheets as acceptors, the fluorescence signal was quenched in the absence of competitive binding from circulating tumor cells. The mechanism was proven faster than most so far (15 min), and it was possible to detect as few as 10 CTCs in whole blood. However, it has been reported that the CTCs had a tendency to engulf the EpCAM@aptamer@Fe_3_O_4_@GQD complexes which may significantly reduce the detection limit of the sensor. 

**Figure 2:**
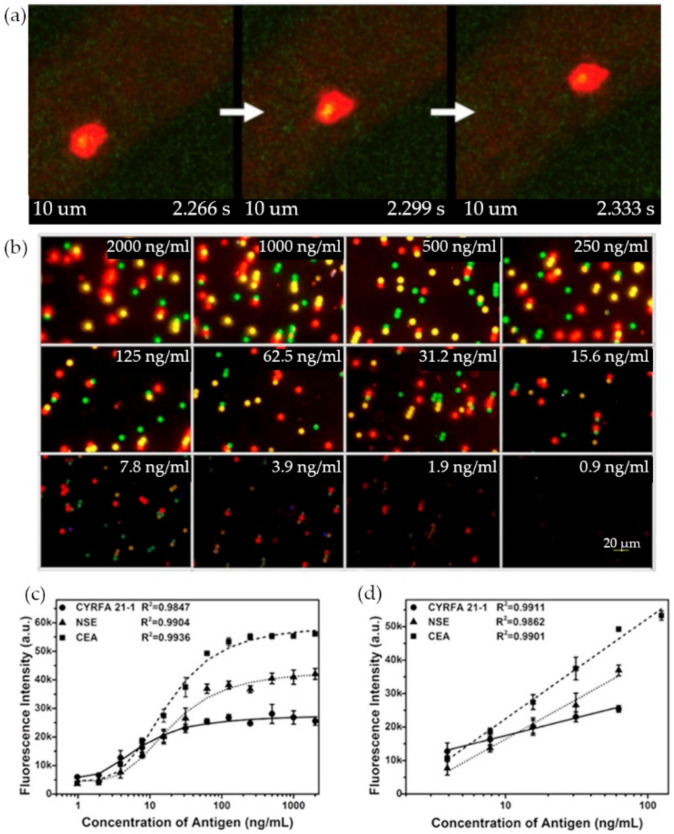
Quantum dots (QDs)-based fluorescence assays: (**a**) Three consecutive images (33 ms interval) of CD133+  circulating tumor cells (CTCs) tagged with red QDs moving in a blood vessel. (**b**) Multiplexed fluorescence of cytokeratin 19 (CYRFA 21-1), neuron-specific enolase (NSE), and carcinoembryonic antigen (CEA) at different concentrations. (**c**) Standard curves for the detection of CYRFA 21-1, NSE, and CEA in the multiplexed assay. (**d**) Linear range of the plot in (**c**). (Reproduced with permission from [20], published by Springer Nature 2019; reproduced with permission from [23], published by Elsevier 2016).

As CTCs tend to travel through the bloodstream to establish new colonies, most experiments are meant to analyze the blood CTCs concentrations to establish a diagnostic for cancer patients. Nonetheless, it seems to be equally important to have a better understanding of the routes those circulating cancer biomarkers take. Kuo et al. used an in vivo approach to CTCs detection [20]. In their experiment, antibody-conjugated QDs were used to track the movement of an animal cancer model CTCs with real-time fluorescent imaging. After inducing a tumor growth in the earlobes of mice from pancreatic cancer cells expressing red fluorescent protein (RFP), CD24 functionalized QDs 525 (Green) were delivered to the mice bloodstream by injection to study the movement of a subpopulation of CTCs through multi-photon microscopy. A more metastatic CTCs subpopulation expressing green fluorescent proteins were also tagged with anti-CD133 antibody linked QDs 705 (Red) to image the movement of a smaller number of cells (Figure 2a) [20].

Due to the fact that our body fluids are interconnected, smaller circulating cancer markers can be found in the urine of the saliva of cancer patients, quantum dot-based nanobiosensors were adapted to different liquid biopsies. In a recent experiment by Nejdl et al., self-assembled cadmium telluride (CdTe) QDs were used for the detection of nucleic acid excreted in the urine [21]. The nanoparticles were synthesized through a simple spontaneous reaction that lasted 70 h. The QDs and a methylene blue quencher were combined for the sensing of DNA. Both the absorbance and the fluorescence were measured and capillary electrophoresis (CE) with laser-induced fluorescence detection was also used [21]. Another study exploring salivary exosome biosensing was based on combined aptamer recognition. A complex was formed between self-assembled DNA concatemer and numerous QDs bound to an aptamers–magnetic microspheres combination. When one exosome binds to the aptamer, it would release many QDs from the DNA concatemer which amplifies the fluorescent signal. The biosensor required a significantly short time of incubation of 30 min [22].

Most biosensors have been targeting a single type of circulating cancer biomarker when in reality it is often the presence of multiple biomarkers that is a better cancer indicator. In a triplex sandwich assay by Wu et al., three QDs and micro-sized magnetic beads pairs were functionalized with monoclonal antibodies that targeted three lung cancer biomarkers in solution: cytokeratin 19 (CYRFA 21-1), carcinoembryonic antigen (CEA), and neuron-specific enolase (NSE) [23]. Each one of the QDs was specifically chosen with a different emission maximum (green—525 nm, yellow—585 nm, and red—625 nm) and photographed with an optical microscope before getting treated by Gray Quantifier software. The nanoprobes allowed for the simultaneous detection and quantification of three analytes (Figure 2b–d) at a low cost, with a limit of detection (LOD) of 364 pg/mL for CYRFA21-1, 38 pg/mL for CEA, and 370 pg/mL for NSE in a sample volume as low as 20 µL [23]. The experiment required an incubation time of 1 h.

Electrochemiluminescence (ECL) is another transduction mechanism by which the potential of QDs has been harnessed in an attempt to improve the detection sensitivity of nanoparticle-based optical biosensors. In an experiment by Meng et al., molybdenum oxide(MoOx) QDs were used in the ECL detection of non-small cell lung cancer biomarker CYFRA21-1 to achieve an even lower LOD of 0.3 pg/mL [24]. A layered detection platform was made by assembling a gold nanoparticles (AuNPs) layer meant to increase the electron transportation rate, a cathodic layer of MoOx QDs, and a layer of target-specific antibodies. By forming a complex with those antibodies, CYFRA21-1 hinders the electron transfer in a concentration-dependent manner and thus reduces the ECL signal. This type of sensor had a good sensitivity. However, full biological samples may potentially affect its detection mechanism due to some crosstalk or a reduced electron transportation rate [24]. 

**Figure 3:**
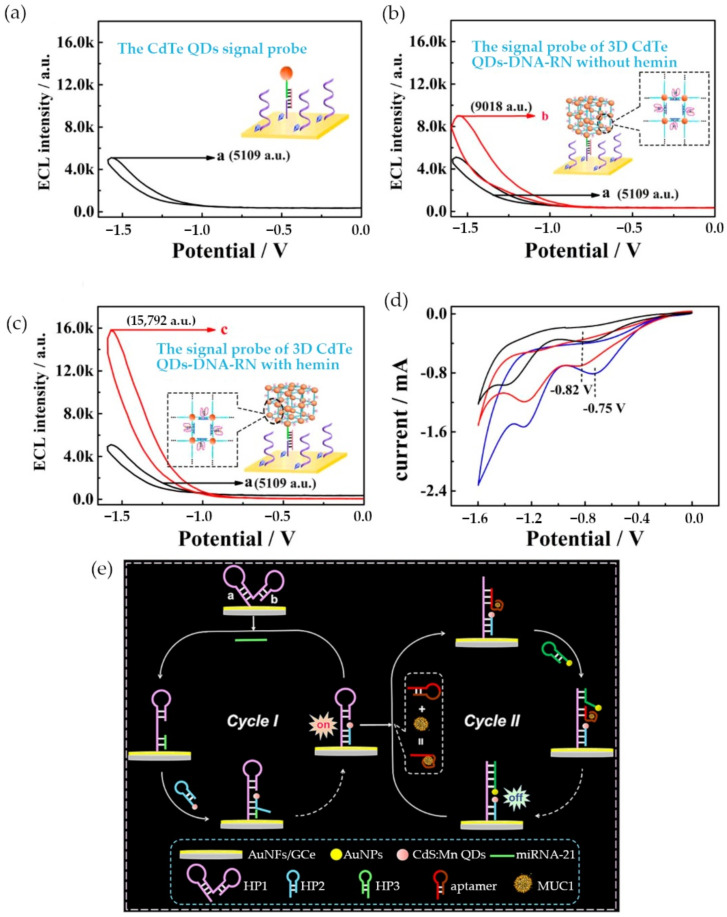
QDs-based electrochemiluminescence (ECL) sensing: (**a**) ECL curve of QDs signal probe; (**b**) ECL curve of 3D QDs-DNA-RN without hemin signal probe; (**c**) ECL curve of 3D QDs-DNA-RN with hemin signal probe; (**d**) cyclic voltammetry responses of the QDs signal probe; (**e**) schematic of the miRNA-21 and MUC1 detection platform. (Reproduced with permission from [26], published by Elsevier 2020; reproduced with permission from [27], published by American Chemical Society 2019).

Mahani et al. developed a biosensor for the detection of ovarian cancer marker miRNA-21 based on a FRET system [25]. The sensor was constructed by a molecular beacon with a carbon QD at one end and a Black Hole Quencher 1 (BHQ1) at the other end. With both ends in close proximity, the fluorescent emission of the QD was quenched [25]. Conversely, in the presence of the exact sequence of miRNA-21, the molecular bacon would go through a conformational change that increased the gap between the QD and BHQ1 which would induce a fluorescent signal. The biosensor was highly specific and had a limit of detection of 0.3 nM [25]. However, during the same year, Sun et al. were able to achieve a lower LOD of 34 aM for miRNA-21 detection by engineered an ECL-based sensor [26]. The team designed a 3D walker probe by linking cadmium telluride (CdTe) QDs with various DNA sequences (Figure 3a–d) [26]. The nanoreticulations of the hemin/G-quadruplex structure increased the availability of QDs, thus when in presence of miRNA-21, the ECL signal was stronger.

A year later, Li et al. achieved a significantly lower limit of detection by developing a two-cycle sensitive platform based on dual catalytic hairpin assembly for the detection of both miRNA-21 and mucin 1 with an LOD of 11 aM and 0.40 fg/mL, respectively [27]. During the first cycle of the platform, miRNA-21 bound to a hairpin structure and initiated the addition of another manganese doped cadmium sulfide (CdS:Mn) QD modified hairpin structure. This led to the emission of an initial ECL signal. The second cycle generated an ECL-RET signal through the binding of mucin 1/aptamer to the CdS:Mn QD modified hairpin structure, which later induced the insertion of AuNPs into the structure that decreased the ECL signal (Figure 3e) [27]. Recent QDs-based optical biosensors for circulating cancer biomarkers are compared in Table 1.

**Table 1:** Quantum dots-based optical biosensors for circulating cancer biomarkers.

Sensing Mechanism	Target Biomarker	Detection Elements	Signal Elements	LOD	Reference
Fluorescence	CTC ^1^ (SK-Br3)	Anti-EpCAM antibodies	Octadecylamine-coated QDs ^2^ 630	275 cells/mL	[18]
	CTC (MCF-7)	Anti-EpCAM antibodies	ZnS-coated CuInSe QDs	12 cells/well	[28]
	Exosomes (CAL27)	CD63 aptamers	ZnCdSe/ZnS core/shell QDs	500 particles/μL	[22]
	CYRFA 21-1 ^3^, CEA ^4^ and NSE ^5^	Target specific antibodies	525, 585 and 625 QDs	364 pg/mL, 38 pg/mL and 370 pg/mL	[23]
PET ^6^	ctDNA ^7^	Semi-intercalation binding with magnetic beads	Mercaptosuccinic acid stabilized CdTe QDs	3 ng/mL	[21]
NSET ^8^	CTC (Hep G2 and A549)	EpCAM aptamers	Nitrogen and sulphur-doped graphene QDs (donors) quenched by MoS_2_ nanosheets (acceptors)	1.19 nM	[19]
FRET ^9^	AFP ^10^	AFP aptamers and anti-AFP antibodies	CdTe QDs (donors) quenched by AuNPs (acceptors)	400 pg/mL	[29]
	miRNA ^11^-21	Hairpin-structured oligonucleotide probes	Carbon QDs (donors) quenched by Black Hole Quencher 1 (acceptors)	0.3 nM	[27]
ECL ^12^	CEA	CEA antibodies	Poly(ethylenimine) functionalized graphene oxide matrix modified with carbon QDs and AuNPs	1.67 pg/mL	[30]
	CYFRA21-1	CYFRA21-1 antibodies	Molybdenum oxide QDs/Au NPs-chit nanocomposite	0.3 pg/mL	[24]
	miRNA-21 and MUC1 ^13^	miRNA-21 hairpin probes and MUC1 aptamers	HP2 ^14^ modified by CdS:Mn QDs and AuNPs modified hairpin probes	11 aM and 0.40 fg/mL	[26]
	miRNA-21	miRNA-21 specific hairpins	3D CdTe QDs–DNA nanoreticulations	34 aM	[25]

^1^ Circulating tumor cell. ^2^ Quantum dots. ^3^ Cytokeratin 19. ^4^ Carcinoembryonic antigen. ^5^ Neuron-specific enolase. ^6^ Photoinduced electron transfer. ^7^ Circulating tumor DNA. ^8^ Nanosurface energy transfer. ^9^ Fluorescence resonance energy transfer. ^10^ Alpha-fetoprotein. ^11^ Micro-RNA. ^12^ Electrochemiluminescence. ^13^ Mucin 1. ^14^ Hairpin 2.

## 3. Metal Nanoparticle-Based Optical Biosensor for Circulating Cancer Biomarkers

In the category of metal nanoparticle-based biosensors, copper, silver, and gold are the most common elements. However, due to the instability of Cu, Au and Ag nanoparticles are predominantly used. Multiple physical, chemical and biological approaches are available for the synthesis of metal nanoparticles. The choice of method depends on different factors such as the time or the cost of synthesis but also the size and the shape of the nanoparticles. Metal nanoparticles are often used in fluorescence assays. However, due to the fact that they are not fluorescent elements by nature, they require grouping with a fluorescent dye. For instance, in the detection of exosomes as circulating cancer biomarkers, Gao et al. used a dual hairpin signal amplification mechanism where gold nanoparticles (AuNPs) only served as a scaffold and where the fluorescent signal was emitted by Fluorescein, an organic dye [31]. In other cases, electrocatalytic features of metal nanoparticles are coupled with ECL essays [32]. However, when it comes to optical biosensors, the most important characteristic of metal nanoparticles is their localized surface plasmon resonance (LSPR). LSPR is a physical phenomenon where the electrons on the surface of a metal NP collectively oscillate after being exposed to an exciting light.

In 2015, Pallares et al. engineered an inverse sensitivity plasmonic nanosensor based on the LSPR of gold nanorods for the quantification of circulating tumor DNA (ctDNA) [33]. Hexadecyltrimethylammonium bromide (CTAB)-coated gold nanorods and ctDNA would aggregate based on their electrostatic interactions. In theory, when the rods are covered by the ctDNA, their UV-visible light spectroscopy signal is reduced in a concentration-dependent manner. The mechanism of detection was simple and sensitive for the detection of lower concentrations, but it had a few stumbling blocks. The first step entailed the extraction of the ctDNA through a commercial kit which may reduce the analyte yield [33]. Another major issue was that ctDNA seemed to induce both aggregation and disaggregation of nanorods at different concentrations. Considering that clinical samples have variable ctDNA concentration, this requires accordingly tune the dynamic ranges of the detection [33]. Recently, Wang et al. developed a detection platform for cancerous exosomes. The mechanism was based on a gold film surface-functionalized with exosome capture DNA strands [34]. When exposed to the biomarker, exosome-specific aptamer and single-strand DNA T_30_ linked AuNPs were added, followed by T_30_ linked AuNPs. After 60 min of incubation time, the coupling of the SPR from the Au film and the LSPR from both types of AuNPs amplified the detection signal of the exosomes with a limit of detection as low as 5 × 10^3^ exosomes/mL [34].

**Figure 4:**
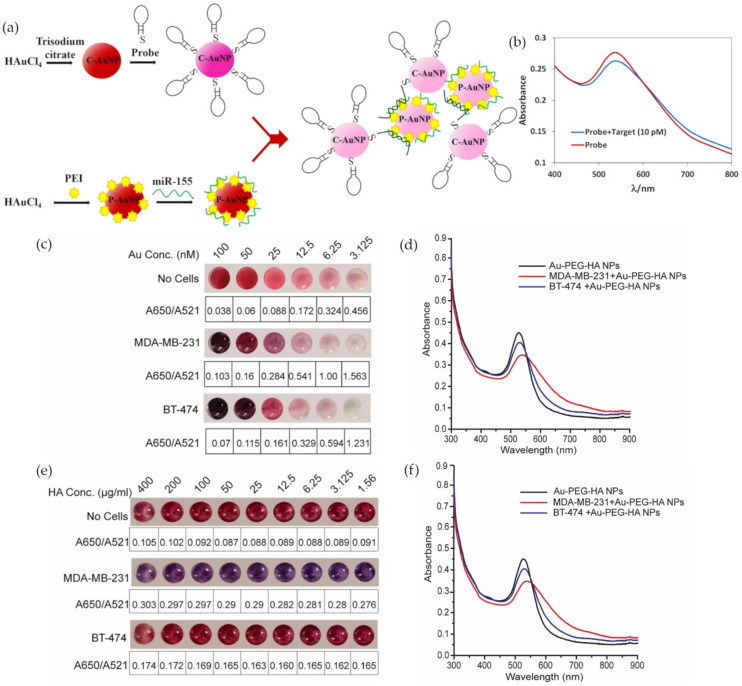
Gold nanoparticle (AuNPs)-based colorimetric assays: (**a**) Schematic of miRNA-155 detection probes; (**b**) UV-Vis spectra curve of the detection probes in the absence (red) and presence (blue) of miRNA-155; (**c**) colorimetric changes and A650/A521 ratio of Au-polyethylene glycol- hyaluronic acid (HA) NPs after incubation with 50,000 cells (MDA-MB-231 and BT-474) at varied Au concentrations; (**d**) UV-Vis spectra of Au-polyethylene glycol-HA NPs after incubation with MDA-MB-231 (50,000 cells) and BT-474 (50,000 cells) at 25 nM Au concentration; (**e**) colorimetric changes and A650/A521 ratio of Au-polyethylene glycol-HA NPs after incubation with MDA-MB-231 (50,000 cells) and BT-474 (50,000 cells) at varied HA concentrations and 25 nM Au; (**f**) UV-Vis spectra of Au-polyethylene glycol-HA NPs after incubation with 50,000 cells (MDA-MB-231 and BT-474) at 100 HA µg/ml and 25 nM Au concentrations. (Reproduced with permission from [35] and from [36], published by Springer Nature 2018).

Metal nanoparticle colorimetric assays are also based on LSPR. When M-NPs aggregate in solution, there is a coupling of their surface plasmon resonance that causes a quick change in color and a shift in the absorbance peak of UV-visible light spectra of the sample. In an attempt to avoid the use of the expensive instruments and the tedious protocols that miRNA and ctDNA testing traditionally require, more researchers are trying to take advantage of this visual sensor. Hakimian et al. devised an ultrasensitive optical biosensor for the detection of breast cancer through an miRNA-155 sensing assay based on crosslinking aggregation [35]. Positively charged polyethyleneimine capped AuNPs (P-AuNPs) were synthesized by thermal reduction to trap negatively charged miRNA-155. A similar type of AuNPs (C-AuNPs) was citrate capped and linked to a thiolated DNA hairpin meant to hybridize with the P-AuNPs/miRNA-155 complex (Figure 4a). The miRNA served as an aggregation promoter between the two types of AuNPs which affected the molar ratio of dispersed to aggregated AuNPs, A_530_/A_750_ (Figure 4b). The sensor specificity was successfully tested for three base-pair mismatches, and it achieved a detection limit of 100 aM within approximately 45 min [35].

As the quest for more sensitive, more accessible, and less time-consuming assays continues, Wang et al. worked on improving colorimetric sensors specifically for the detection of single-nucleotide polymorphisms (SNPs) in ctDNA. The detection assay was optimized through various methods to achieve an LOD of 67 pM [37]. For instance, a critical linker concentration had to be determined and an extra centrifugation step had to be taken to ease the visual detection of KRAS oncogene mutations [37]. Rauta et al. went even further by developing a CTCs detection platform that takes CTCs viability into account [36]. AuNPs were synthesized and added to polyethylene glycol before getting functionalized with hyaluronic acid (HA), a ligand to CD44 expressing cancer cells. Different concentrations of Au and HA were tested with two cell lines that express variable levels of CD44 to find the optimal values for the nanoprobe (Figure 4c–f), but further optimization for a uniform distribution of HA is needed. The cell viability of the detection platform was between 60 and 94%, promising numbers for additional assays [36].

**Figure 5:**
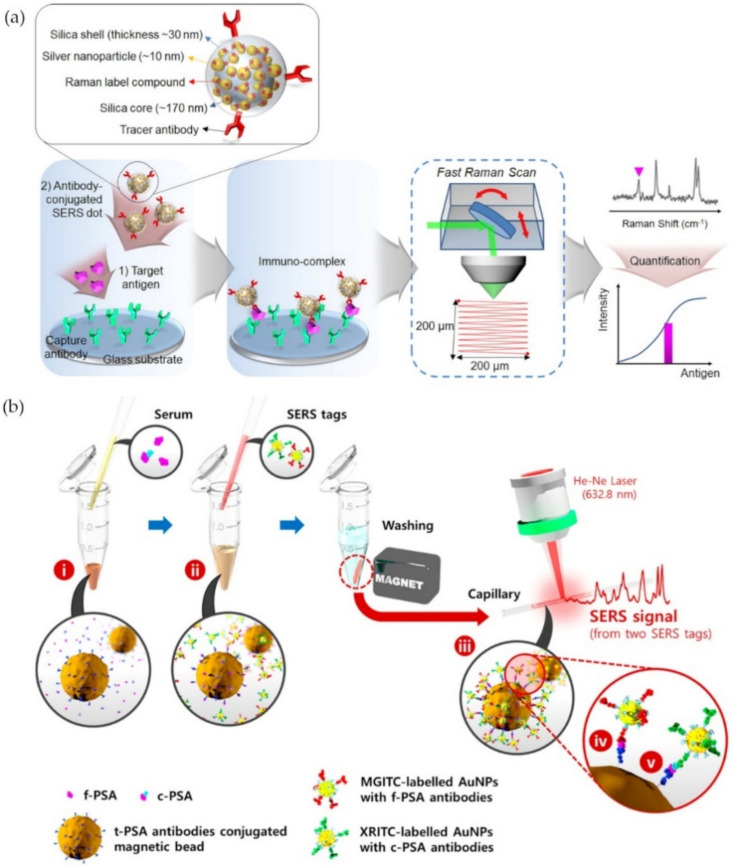
Schematics of surface-enhanced Raman scattering (SERS) assays for the detection of the prostate-specific antigen (PSA): (**a**) Simultaneous detection of free PSA and complexed PSA; (**b**) SERS signal area scanning for PSA detection. (Reproduced with permission from [38], published by American Chemical Society 2016; reproduced with permission from [39], published by American Chemical Society 2017).

Surface-enhanced Raman scattering (SERS) is another optical transduction mechanism that measures the light scattering caused by the LSPR property of metal nanoparticles. The coating of silver nanoparticles by Raman reporter molecules on a silica core followed by their encapsulation in a silica shell that is then conjugated with secondary antibodies produced nanoparticle probes that Chang et al. named SERS dots [38]. These probes were used in a sandwich-type immunoassay to target the prostate-specific antigen (PSA) bound to an antibody-immobilized glass substrate in an attempt to scan the Raman spectra of the whole area all at once (Figure 5a). This SERS detection method was highly sensitive with an achieved LOD of 3.4 fM [38]. In another SERS-based immunoassay (SIA), gold nanoparticles loaded with Raman report molecules and functionalized with free or complexed PSA antibodies formed a sandwich immunocomplex with a total-PSA antibody conjugated magnetic bead in the presence of PSA from clinical serum samples (Figure 5b) [39]. The purpose of the experiment was to measure the ratio of free to total PSA for a more accurate diagnosis as a higher total PSA level and a lower percentage of free PSA are associated with a higher risk of prostate cancer. Cheng et al. were able to design a fast assay, less than 1 h, for the simultaneous detection of dual PSA markers with an LOD as low as 0.012 ng/mL for free PSA and 0.15 ng/mL for complexed PSA (c-PSA) [39].

However, the usage of specific antibodies could be disadvantageous due to their time-consuming and costly production. Furthermore, antibodies are prone to degradation due to their instability. To overcome this problem, Yang et al. used a magnetic aptasensor to detect and separate PSA from human serum samples [40]. Satellite AuNPs were functionalized with PSA-complementary DNA and bound to a core magnetic nanoparticle that was functionalized with PSA-aptamers. In the presence of PSA, the gold nanoparticles would be separated from the complex due to competitive binding and by doing so, once the magnetic NPs were removed from the solution by a magnet, the SERS signal was amplified with an LOD of 5.0 pg/mL [40].

In a similar way to SERS, the LSPR of metal NPs plays a role in metal enhanced fluorescence (MEF) by increasing the excitation state of a fluorophore. Doing so, the optical proprieties of the dye are changed: a higher quantum yield, better stability, and a shorter lifetime [41]. Based on this mechanism, Xu et al. were able to develop an immunoassay for the detection of PSA reaching an LOD of 27 pg/mL [42]. The experiment, based on Silica coated AgNPs combined with RuBpy fluorescent particles, required a total of 30 min. In another experiment by Choi et al., the LSPR of Ag nanocubes was used to enhance Alexa-488 fluorescence and achieve an LOD of 1 ng/mL for CEA [43]. Recent metal nanoparticles-based optical biosensors for circulating cancer biomarkers are compared in Table 2.

**Table 2:** Metal nanoparticle-based optical biosensors for circulating cancer biomarkers.

Sensing Mechanism	Target Biomarker	Detection Elements	Signal Elements	LOD	Reference
SPR ^1^	Exosome (MCF-7)	CD63 aptamers	Au film, aptamer/T_30_ linked and A_30_ linked AuNPs ^2^	5 × 10^3^ exosomes/mL	[34]
	miRNA ^3^-21 and CTC ^5^ (SMMC-7721)	Hairpin probes and cell-specific aptamers	Au film, DNA-linked AuNPs, and AgNPs ^4^	0.6 fM and 1 cell/µL	[44]
	ctDNA ^6^	Electrostatic interactions	Hexadecyltrimethylammonium bromide coated Au nanorods	0.2 nM	[33]
Colorimetry	CTC (MDA-MB-231)	CD44 ligands	AuNPs-conjugated hyaluronic acid	N/A	[36]
	Exosome (C666-1)	Target specific antibodies	AuNP–DNA conjugates	100 particles/mL	[45]
	Flt-1 ^7^	Target specific ligand peptides	Peptide-coated AuNPs	0.2 nM	[46]
	miRNA-155	Hairpin DNA probes	Citrate-capped and polyethyleneimine-capped AuNPs	100 aM	[35]
	ctDNA (KRAS)	Complementary linkers	DNA oligonucleotides–functionalized AuNPs	67 pM	[37]
SERS ^8^	CTC(HeLa and MCF-7)	Targeted specific ligand folic acid	Reductive bovine serum albumin-stabilized AuNP coated with 4-mercaptobenzoic acid	5 cells/mL	[47]
	Exosome (SKBR3, T84, and LNCaP)	H2, CEA, and PSMA aptamers	5,5′-dithiobis(2-nitrobenzoic acid), 2-naphthalenethiol or 7-mercapto-4-methylcoumarin labeled AuNPs	32, 73, and 203 exosomes/µL	[48]
	PSA ^9^	Target specific antibodies	Raman label compound coated AgNPs bound to a silica core	0.11 pg/mL	[38]
	PSA	PSA aptamers	4,4′-dipyridyl-labeled AuNPs	5.0 pg/mL	[40]
	free-PSA and complexed-PSA	Target specific antibodies	Malachite green isothiocyanate and/or X-rhodamine-5-(and-6)-isothiocyanate labeled AuNPs	0.012 ng/mL and 0.15 ng/mL	[39]
MEF ^10^	PSA	Target specific antibodies	Silica-coated AgNPs and RuBpy	27 pg/mL	[42]
	CEA ^11^	Target specific antibodies	Ag nanocubes and Alexa-488	1 ng/mL	[43]

^1^ Surface plasmon resonance. ^2^ Gold nanoparticles. ^3^ Micro-RNA. ^4^ Silver nanoparticles. ^5^ Circulating tumor cell. ^6^ Circulating tumor DNA. ^7^ Vascular endothelial growth factor receptor 1. ^8^ Surface-enhanced Raman scattering. ^9^ Prostate-specific antigen. ^10^ Metal enhanced fluorescence. ^11^ Carcinoembryonic antigen.

## 4. Upconversion Nanoparticle-Based Optical Biosensor for Circulating Cancer Biomarkers

Upconversion nanoparticles (UCNPs), principally from the lanthanide group, have good autofluorescence inhibition and deep tissue penetration which makes them excellent candidates in the biosensing field. The most interesting feature of UCNPs is their capacity to upconvert low-energy photons to high-energy photons when exposed to infrared light (700–1000 nm). By doing so, they can emit ultraviolet or visible lights after excitation with a 980 nm light, the most commonly used type of laser. UCNPs are composed of multiple elements: a host lattice that serves as a conducting structure, doped with activator and sensitizer ions for the transfer of energy [49]. It is also possible to change the UCNPs emitted light by changing their dopants, thus opening a door for potential multiplexing assays. It is possible to synthesize UCNPs through a few methods, namely, thermal decomposition, hydrothermal synthesis, or chemical co-precipitation [50]. Once synthesized it is possible to functionalize them and used them as sensing nanoprobes for the detection of circulating cancer biomarkers.

**Figure 6:**
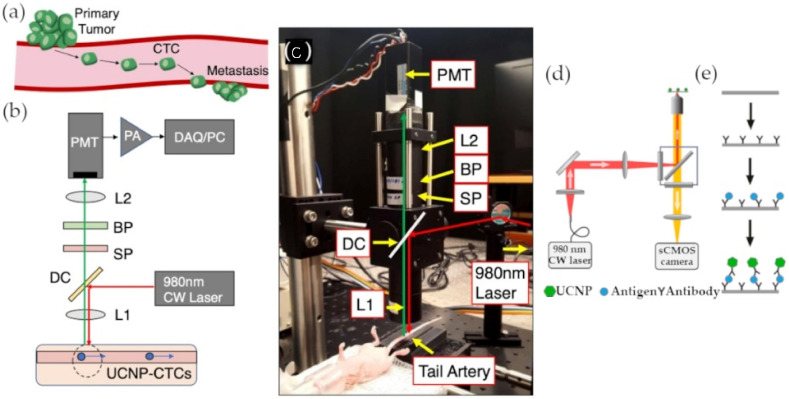
Upconversion nanoparticle (UCNP) detection settings. (**a**) Schematic of primary tumor metastasis; (**b**) schematic of in vivo CTCs detection by diffuse in vivo flow cytometry (U-DiFC); (**c**) photograph of U-DiFC setting; (**d**) schematic of in vitro circulating cancer biomarker detection setting; (**e**) schematic of UCNP sandwich immunoassay. (Reproduced with permission from [52], published by Dove Medical Press Limited 2020; reproduced with permission from [53], published by American Chemical Society 2017).

In a recent experiment by Guo et al., epithelial cell adhesion molecules (EpCAMs) were once again used as a target for the detection of circulating tumor cells (CTCs) [51]. NaEuF_4_-type UCNPs were synthesized through solid-liquid thermal decomposition and functionalized with anti-EpCAM antibodies. Microplate wells were also decorated with the same antibodies to retain only the EpCAM-expressing MCF-7 breast cancer cells from whole blood samples. The detection protocol combined a dissolution enhanced luminescence method, involving an enhancer solution that increased the release of Eu^3+^ from the UCNPs, with the measurement of a time-resolved photoluminescence (TRPL) signal where the intensity of the signal spike depended on the number of cancer cells. By doing so, it was possible to overcome the low concentration of CTCs by increasing the fluorescence of the UCNPs and neglecting the autofluorescence interference caused by other elements in the whole blood sample by delaying the TRPL signal reading. The nanoprobe achieved a limit of detection as low as 1 cell/well by utilizing UCNPs’ unprecedented optical properties [51]. In an attempt to exploit the low toxicity of lanthanide-based UCNPs, another advantage of the nanoparticles, Bartosik et al. developed a new laboratory-made instrument for the detection of CTCs named UCNP-compatible diffuse in vivo flow cytometry (U-DiFC) [52]. The instrument’s purpose is to measure the fluorescence emission of UCNPs from the tail artery of a mouse without any puncture site. The signal had to pass through multiple lenses and filters before getting amplified by a pre-amplifier and digitized by a multi-function data acquisition board (Figure 6a–c). Despite the promising results of the cells co-incubated with UCNPs and tested on an optical flow phantom model, the CTCs were not internalizing the UCNPs in vivo as a contrast agent should [52]. Nonetheless, the failure of the assay did not undermine the potential of UCNPs-based fluorescence detection of circulating cancer biomarkers.

**Figure 7:**
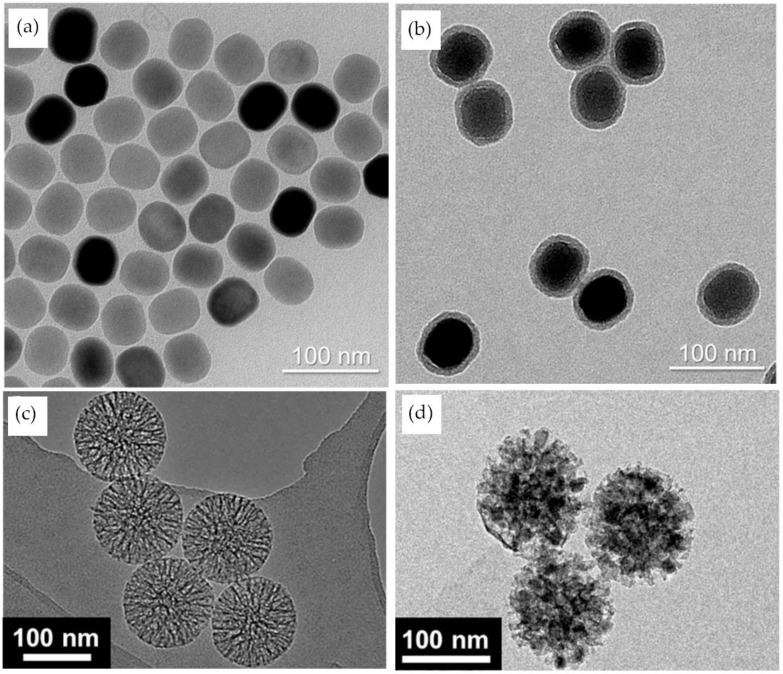
Combination of UCNPs with a silica core or shell: (**a**) TEM image of oleic acid-capped UCNPs; (**b**) TEM image of UCNPs with carboxylated silica shell; (**c**) SEM of mesoporous silica nanoparticles; (**d**) TEM image of CaF_2_: RE^3+^ UCNPs embedded mesoporous silica nanoparticles. (Reproduced with permission from [53], published by American Chemical Society 2017; reproduced with permission from [55], published by Elsevier 2019).

As the detection of other circulating biomarkers can occur earlier than the detection of circulating tumor cells, another team of researchers (Lan et al.) focused on developing an assay for the quantitative detection of vascular endothelial growth factor (VEGF), a circulating protein that has an increased concentration in breast cancer patients [54]. They used the thermal decomposition of rare-earth stearates to synthesize cheap and pollution-free NaYF_4_:Yb^3+^, Er^3+^ nanoparticle. They also designed a VEGF-specific aptamer that was divided into two portions. The UCNPs were then functionalized with a portion of the VEGF-specific aptamer, and the other portion was fixed on a 96-well microplate. By doing so, the two portions of the aptamer would form a complex only in the presence of the protein of interest and subsequently produce a 540 nm luminescent signal when excited by a 980 nm light [54]. Lan et al. achieved an LOD of 6 pM for the detection of VEGF, in breast cancer, but with a similar experiment, Farka et al. were able to lower the LOD to 42 fM for PSA, a circulating protein in prostate cancer (Figure 6d,e) [53]. The biosensor was based on a single molecule upconversion-linked immunosorbent assay (ULISA). After synthesizing β-NaYF_4_:18 mol % Yb^3+^, 2 mol % Er^3+^ type UCNPs by high-temperature coprecipitation, the particles were coated with silica (Figure 7a,b) and functionalized with anti-PSA antibodies. The same antibodies were also fixed on a microtiter plate to form a sandwich immunoassay in the presence of the PSA protein and the UCNPs-based detection probe. The number of complexed UCNPs was then automatically counted by an upconversion epiluminescence microscopy software [53].

It is often the combinatory analysis of multiple biomarkers that is relevant in cancer biology. Herein, to be able to simultaneously quantify the presence of multiple types of miRNA, Gu et al. designed a fluorescence detection method based on mesoporous silica nanoparticles embedded with upconversion nanocrystals (UCNCs) [55]. Before forming the nanocrystals by thermo-decomposition, a precursor had to be introduced into the mesoporous silica particles through a capillary effect (Figure 7c,d). To be able to detect multiple targets in the same sample, different lanthanide dopants, Tm^3+^ or Ho^3+^, were used in a combination with different DNA sequences to target breast-related miRNA-195 and miRNA-21. The experiment was successful in establishing a correlation between the fluorescence of the nanoprobes and the variable concentration of a mixture of miRNAs with a reported LOD of 100 nM for miRNA-195 [55]. However, despite the multiplexing potential of this assay, a significantly lower limit of detection was achieved by the UCNPs FRET-based sensors.

UCNPs are frequently used in fluorescence resonance energy transfer assays due to the fact that they are excellent energy donors. To exploit that quality of UCNPs, Chen et al. constructed a paper-supported biosensor for the detection of exosomes [56]. The aptameric sequence of CD63, a protein expressed on the surface of exosomes, was divided into two portions. One portion was attached to the UCNPs (energy donors) and then bound to a filter paper. The other portion was linked to Au nanorods (energy receptors). In the absence of the exosome, the UCNPs would produce a green fluorescence when exposed to a 980 nm light. However, in the presence of the exosome, both fragments, and subsequently nanoparticles, would be brought together, causing the quenching of the fluorescence proportional to the number of exosomes with an LOD of 1.1 × 10^3^ particles/µL within approximately 30 min [56]. A year later, Wang et al. were able to achieve a significantly lower LOD of 80 particles/µL with another paper-based aptasensor for the detection of exosomes [57]. In this experiment, an EpCAM aptamer was divided between an energy donor (UCNPs) and an energy receptor (tetramethylrhodamine (TAMRA)). Due to the energy transfer between both particles, TAMRA emits a yellow fluorescence. By quantifying TAMRA’s emissions, it was possible to determine the concentration of EpCAM expressing exosomes [57]. In another assay by Wang et al., it was possible to achieve an LOD of 6.30 pM for ctDNA by using gold nanocages as energy acceptors [58]. The UCNPs were designed to competitively bind to ctDNA and free themselves from the quenching effect of the gold nanocage. Emerging materials such as graphene oxide are also demonstrating promising results in the field. A UCNP-based DNA biosensor, where graphene oxide was used as an energy acceptor, achieved an LOD of 5 pM [59]. A similar sensor by Vilela et al. demonstrated an LOD of 500 fM for PCA3 miRNA, a prostate cancer biomarker, detection [60]. Both sensors took advantage of the quenching property and the energy acceptor potential of graphene oxides. Recent upconversion nanoparticles-based optical biosensors for circulating cancer biomarkers are compared in Table 3.

## 5. Conclusions

This review covered the most recent advancements in nanoparticle-based optical sensors for the detection of circulating cancer biomarkers. Nanoparticles have repeatedly proven their potential to overcome some of the most difficult aspects of liquid biopsy analysis. Their surface-to-volume ratio, multiplexing capacity, and stable optical proprieties are considerable advantages in the biosensing field (Table 4). However, bearing in mind the complexity of most biological fluids, it is still difficult to achieve both high sensitivity and specific targeting of biomarkers due to multiple factors, such as sample autofluorescence and immunoassay crosstalk, among other things.

Although the current research focuses mostly on the development of new mechanisms of detection and on improving the LOD of the existing ones, a shift is to be expected in the near-future towards more point-of-care-oriented sensors, thus making cancer diagnostic easier, less time consuming, and potentially more affordable to the general population. In vivo fluorescence imaging of circulating cancer biomarkers is also a promising development for the future of optical nanobiosensors, but this will require extended studies on the cytotoxicity of the nanoparticles. For now, the focus will be not only on easing the synthesis and the functionalization of those particles but also on improving their fluorescence by increasing their quantum yield or combining other enhancing mechanisms. 

## Figures and Tables

**Table 3:** Upconversion nanoparticle-based optical biosensors for circulating cancer biomarkers.

Sensing Mechanism	Target Biomarker	Recognition Elements	Signal Elements	LOD	Reference
Fluorescence	CTC ^1^ (MCF-7)	Anti-EpCAM antibodies	NaEuF_4_ UCNPs ^2^	≥10 cells/mL	[53]
	VEGF ^3^	Target specific aptamers	α-NaYF_4_:Yb^3+^, Er^3+^ UCNPs	6 pM	[55]
	PSA ^4^	Anti-PSA antibodies	β-NaYF_4_:18 mol % Yb^3+^, 2 mol % Er^3+^ UCNPs	42 fM	[52]
	miRNA ^5^-195 and miRNA-21	oligonucleotide probes	CaF_2_: RE^3+^ upconversion nanocrystals doped with Ho^3+^, Tm^3+^ or Yb^3+^	100 nM	[54]
FRET ^6^	Exosome (MDA-MB-231 and MCF-7)	EpCAM aptamer	NaYF_4_:Yb,Er UCNPs (donors) and Tetramethyl rhodamine (acceptors)	80 particles/µL	[57]
	Exosome (HepG2)	CD63 aptamer	NaYF_4_:Yb, Er UCNPs (donors) and Au nanorods (acceptors)	1.1 × 10^3^ particles/µL	[56]
	CA125	CA125 aptamer	Polyacrylic acid coated NaYF_4_:Yb,Er UCNPs (donors) and carbon dots (acceptors)	9 × 10^−3^ U/mL	[61]
	CA125	Anti-CA125 antibodies	PEI coated NaYF_4_:Yb,Tm UCNPs (donors) and AgNPs ^7^ (acceptors)	120 pg/mL	[62]
	PSA	Anti-PSA antibodies	NaYF_4_:Yb^3+^, Er^3+^ UCNPs (donors) and AuNPs ^8^ (acceptors)	2.3 pM	[63]
	PSA	Anti-PSA antibodies	NaYF_4_:Yb^3+^,Er^3+^ and NaYF_4_:Yb^3+^,Er^3+^@NaYF_4_:Yb^3+^,Nd^3+^ (donors) and AuNPs (acceptors)	0.01 ng/mL	[64]
	CEA ^9^	Anti-CEA antibodies	NaYF_4_:Yb,Tm UCNPs (donors) and Fluorescein (acceptor)	0.89 ng/mL	[65]
	CEA	CEA aptamer	UCNPs (donors) and graphene oxide (acceptors)	7.9 pg/mL	[66]
	PCA3 miRNA	Oligonucleotide probes	NaYF_4_:Yb,Er UCNPs (donors) and graphene oxide (acceptors)	500 fM	[60]
	ctDNA ^10^ (KRAS)	Oligonucleotide probes	NaYF_4_:Yb:Tm UCNPs (donors) and Au nanocages (acceptors)	6.30 pM	[58]
	Single strand DNA	Oligonucleotide probes	SiO_2_ coated NaYF_4_:Yb,Er UCNPs, (donors) and graphene oxide (acceptors)	5 pM	[59]

^1^ Circulating tumor cell. ^2^ Upconversion nanoparticles. ^3^ Vascular endothelial growth factor. ^4^ Prostate-specific antigen. ^5^ Micro-RNA. ^6^ Fluorescence resonance energy transfer. ^7^ Silver nanoparticles. ^8^ Gold nanoparticles. ^9^ Carcinoembryonic antigen. ^10^ Circulating tumor DNA.

**Table 4:** Advantages and disadvantages of metal nanoparticles, quantum dots and upconversion nanoparticles.

Nanoparticle Type	Advantages	Disadvantages
Metal nanoparticles	Multiple shapes Conductivity Localized surface plasmon resonance Good biological affinity Good energy acceptor	No fluorescence
Quantum Dots	High quantum yield Broadband excitation Multiplexing Size-tunable fluorescence High photobleaching threshold	Toxicity Blinking effect
Upconversion nanoparticles	Anti-Stokes luminescent Autofluorescence inhibition Multiplexing High chemical stability Deep tissue penetration Dopants-tunable fluorescence	Toxicity Restricted Quantum Yield

## Data Availability

Data sharing not applicable to this article.
